# Impaired Contingent Attentional Capture Predicts Reduced Working Memory Capacity in Schizophrenia

**DOI:** 10.1371/journal.pone.0048586

**Published:** 2012-11-12

**Authors:** Jutta S. Mayer, Keisuke Fukuda, Edward K. Vogel, Sohee Park

**Affiliations:** 1 Department of Psychology, Vanderbilt University, Nashville, Tennessee, United States of America; 2 Department of Psychology, University of Oregon, Eugene, Oregon, United States of America; Institute of Automation, Chinese Academy of Sciences, China

## Abstract

Although impairments in working memory (WM) are well documented in schizophrenia, the specific factors that cause these deficits are poorly understood. In this study, we hypothesized that a heightened susceptibility to attentional capture at an early stage of visual processing would result in working memory encoding problems. 30 patients with schizophrenia and 28 demographically matched healthy participants were presented with a search array and asked to report the orientation of the target stimulus. In some of the trials, a flanker stimulus preceded the search array that either matched the color of the target (relevant-flanker capture) or appeared in a different color (irrelevant-flanker capture). Working memory capacity was determined in each individual using the visual change detection paradigm. Patients needed considerably more time to find the target in the no-flanker condition. After adjusting the individual exposure time, both groups showed equivalent capture costs in the irrelevant-flanker condition. However, in the relevant-flanker condition, capture costs were increased in patients compared to controls when the stimulus onset asynchrony between the flanker and the search array was high. Moreover, the increase in relevant capture costs correlated negatively with working memory capacity. This study demonstrates preserved stimulus-driven attentional capture but impaired contingent attentional capture associated with low working memory capacity in schizophrenia. These findings suggest a selective impairment of top-down attentional control in schizophrenia, which may impair working memory encoding.

## Introduction

Impairments in working memory (WM) are regarded as a fundamental cognitive deficit in schizophrenia [1]. WM refers to the short-term storage of information in the service of the active guidance of behavior [2]. It is crucial for a broad range of cognitive operations, and WM impairments can lead to deficits in social and occupational functioning [3]. Spatial WM deficits are present in high-risk populations [4], in spectrum disorders [5], and in unaffected relatives [6] and therefore have been discussed as a potential endophenotypic marker for schizophrenia [7]. Although WM deficits are well documented in schizophrenia, the specific factors that cause these deficits are not yet known.

Considerable evidence suggests that the capacity of visual WM is restricted to about three or four organized chunks [8], [9]. Nevertheless, stable and substantial differences in WM capacity can be found across healthy individuals [10], [11], which may reflect poor functioning of attentional control in the service of WM [11], [12]. For instance, healthy individuals with low WM capacity show reduced selectivity during WM encoding and as a consequence, may store task-irrelevant information, whereas those with high WM capacity efficiently filter out irrelevant information [11]. The selection of relevant information to be stored in WM is associated with activitv in the prefrontal cortex and the basal ganglia and excerts control over the parietal cortex where the information is stored [13], [14]. Moreover, recently it has been shown that the time needed for healthy individuals to disengage from a distracting event is related to WM capacity [15].

Although there is considerable evidence that low WM capacity in healthy participants is associated with inefficient attentional selection, it is less clear whether a failure of attentional selection of incoming information at an early stage of processing also contributes to the severe impairments in WM observed in patients with schizophrenia (PSZ).

Attentional abnormalities have long been thought to be a central feature of schizophrenia [16], however its role in explaining their severe WM impairments has not been resolved yet. To determine whether poor WM in PSZ stems from problems of attentional selection at an early stage of processing, it is necessary to disentangle the specific types of attentional mechanisms at specific stages of processing in schizophrenia [17]. Attentional selection is required when a subject is confronted with competing incoming stimuli and needs to restrict processing to a subset [18]. Several studies revealed that the selection process is intact in PSZ when the relevant stimuli are salient, having a bottom-up processing advantage [19]–[21]. Similarly, PSZ are unimpaired in their ability to select relevant among irrelevant stimuli for WM storage, when salient cues are given [22]. However, when the selection process requires a high degree of top-down control, performance in PSZ is markedly reduced. For instance, the search time per item is significantly increased in PSZ when the target in a visual search task is embedded among highly similar distractors [19]–[21], [23], [24]. Recent evidence suggests that impairments in top-down driven attentional selection occur not only on the level of perceptual processing but also on the level of WM encoding. For instance, in the presence of highly distracting stimuli, PSZ were impaired in their ability to efficiently select task-relevant items for WM encoding [25].

In the present study, we investigated the relationship between impairments in WM and top-down control vs. bottom-up, stimulus-driven processes in attentional selection within one paradigm, i.e. the attentional capture paradigm. The basic mechanisms underlying attentional capture and its relation to WM capacity have been revealed in studies on healthy participants [15], [26]. However, it is largely unclear to what degree these mechanisms are impaired in PSZ and whether the potential impairment is related to the patients' reductions in WM capacity.

To determine WM capacity we used a visual change detection task that has been extensively used in studies of visual WM in healthy participants [8], [10], [11], [15]. The attentional capture paradigm that we used was developed by Fukuda and Vogel [15]. This task allows us to test the ability to resist interference from a distractor under conditions that require either high or low demands on top-down attentional control. The first step of this task involves a visual search which requires subjects to briefly view an array of four colored Landolt Cs that are presented within placeholders for a specific target item. Subjects are asked to report the orientation of the single item that has the target color. On some trials, a task-irrelevant colored box (flanker) is briefly presented flanking one of the placeholders. The flanker could either match the color of the target item (relevant-flanker condition) or appear in a different color (irrelevant-flanker condition). In the irrelevant-flanker condition, attention would be captured automatically and involuntary depending only on the relative saliency of the physical properties of the flanker such as its sudden onset (stimulus-driven capture) [27], [28]. As a result, visual search times are likely to be increased reflecting additional costs on processes needed to disengage from the salient stimulus feature [15].

In the relevant-flanker condition, when the flanker appears suddenly and its color matches the color of the target, top-down driven effects in addition to stimulus-driven effects are likely to contribute to the capture effect and lead to a further increase in visual search times [15]. According to the attentional capture hypothesis [29], [30], the observer would have adopted a top-down attentional set and stimuli that match this set would capture attention (contingent capture). Thus, the contingent capture effect is thought to depend critically on the observer's intentions and goals (i.e., top-down processes), rather than the physical properties of the stimulus per se (i.e., bottom-up processes) [29] but an alternative interpretation is also possible [31]. To isolate top-down driven effects in the present paradigm we subtracted response accuracy in the relevant-flanker condition from response accuracy in the irrelevant-flanker condition (see Methods).

Typically, in visual search tasks, PSZ show increased search times regardless of distractor type and set size [19]–[21], [23]. This finding may reflect a general deficit in the speed of cognitive processing [32] rather than a specific deficit in the attention process. To account for general cognitive slowing, we implemented a staircase procedure in the present task [15]. This allowed us to equate task difficulty in the no-flanker condition by individually establishing the presentation time of the search array needed to reach a criterion.

Taking individual differences in the processing speed into account, we expected that PSZ would exhibit increased susceptibility to contingent attentional capture but not stimulus-driven attentional capture. Moreover, we predicted an inverse relationship between deficits in the contingent attentional capture task and WM capacity in PSZ. This would be taken as evidence that reduced resistance to interference from distractors at an early stage of processing results in WM encoding problems later, thus pointing to an attention-based account of the WM deficits in schizophrenia.

## Methods

### Change Detection Task

#### Participants

Thirty outpatients with schizophrenia (*n* = 23) or schizoaffective disorder (*n* = 7) (PSZ) participated. Diagnoses were made according to Diagnostic and Statistical Manual of Mental Disorders, Fourth Edition (DSM-IV) criteria [33] using structured clinical interviews. The Brief Psychiatric Rating Scale (BPRS) [34], the Scale for the Assessment of Negative Symptoms (SANS) [35], and the Scale for the Assessment of Positive Symptoms (SAPS) [36] were used to assess symptoms (see Table 1).

**Table 1:** Group demographics and clinical information.

	Patients with schizophrenia	Controls (Change Detection)	Controls (Attentional Capture)
	Mean (SD)	Mean (SD)	Mean (SD)
Age	40.6 (8.4)	37.0 (9.1)	37.4 (9.1)
Age range	25–55	24–56	24–56
Female/male	12/18	13/15	11/17
AA ∶ A ∶ C ∶ O	19 ∶ 1 ∶ 10 ∶ 0	8 ∶ 1 ∶ 17 ∶ 2	7 ∶ 1 ∶ 18 ∶ 2
Handedness^a^	53.7 (61.4)	71.6 (53.8)	66.3 (61.2)
Education	13.7 (2.6)	15.2 (2.4)	15.4 (2.3)
IQ^b^	103.2 (8.9)	106.1 (6.6)	106.0 (7.4)
CPE^c^, mg/day	382.9 (392.90)	n/a	n/a
Duration of illness (years)	18.1 (10.2)	n/a	n/a
BPRS	13.4 (7.1)	n/a	n/a
SAPS	14.6 (9.6)	n/a	n/a
SANS	25.5 (12.9)	n/a	n/a
SPQ	n/a	9.1 (7.1)	8.9 (6.7)

AA, African American; A, Asian; C, Caucasian; O, Other; CPE, Chlorpromazine equivalent.

a Measured with the Edinburgh Handedness Inventory.

b Measured with the National Adult Reading Test.

c Twenty-eight patients were medicated, 3 with a first-generation antipsychotic and 23 with a second-generation antipsychotic (11 in combination with mood-stabilizing and/or anxiolytic medication). Two patients received mood-stabilizing medication only. Medications were stable for a minimum of 4 weeks prior to testing. CPEs for two patients who were treated with Iloperidone and Asenapine are not included.

Twenty-eight healthy control subjects (CO) without a history of DSM-IV Axis 1 disorders and no family history of psychosis were recruited from the community. CO were medication-free and screened to rule out schizotypal personality using the Schizotypal Personality Questionnaire (SPQ) [37]. PSZ and CO were matched for age (*t*_56_ = 1.56, *P* = .13), IQ (*t*_56_ = −1.41, *P* = .16), and handedness (*t*_56_ = −1.18, *P* = .24). Years of education were lower in PSZ than CO (*t*_56_ = −2.33, *P*<.05) (Table 1). All subjects had normal or corrected-to-normal vision. Exclusion criteria were a history of head injury, neurological disorder or substance abuse in the six months preceding the study. All subjects gave written informed consent approved by the Vanderbilt University Institutional Review Board (IRB) and were paid.

#### Stimuli, Task, and Procedure

**Figure 1:**
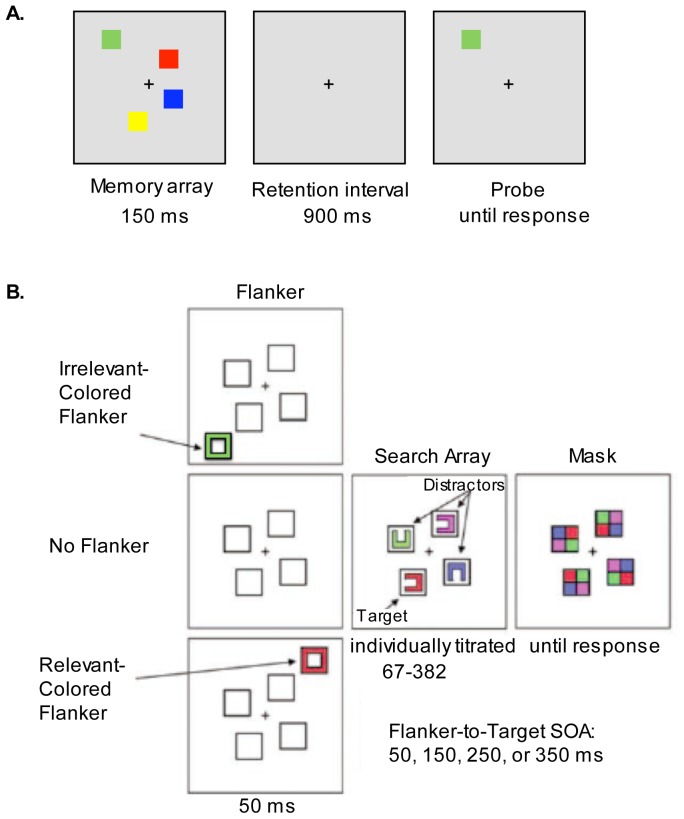
Schematic Diagram of the Procedure and Stimuli in the Experimental Tasks. In the change detection task (A), participants were presented with arrays of two, four, six, or eight colored squares (memory array). After a retention interval they indicated whether the color of the test probe matched or did not match the color of the original memory item in that location. In the attentional capture task (B), participants reported the orientation of the Landolt C that had been presented in the target color (red). In one third of the trials, a flanker (a task-irrelevant colored box) was presented before the search array; in two thirds of the trials, no flanker was presented. In half of the flanker-present trials the flanker was drawn in the target color (relevant-colored flanker), in the other half the flanker was green (irrelevant-colored flanker). The stimulus onset asynchrony (SOA) between the flanker and the search array varied across trials. The duration of the search array was titrated for each participant.

Stimuli in the change detection task were colored (red, green, blue, yellow, purple, black, and white) squares (1.2°×1.2°), presented in randomly selected positions within a centered 11.4°×11.4° region on a gray background (see Figure 1).

In each trial, participants were presented with arrays of two, four, six, or eight colored squares for 150 ms (memory array). After a retention interval of 900 ms, one colored square (test probe) was presented at the location of one of the items from the memory array. Participants made an unspeeded button press to indicate whether the color of the test probe matched or did not match the color of the original memory item in that location. Half of the trials were matches. An inter-trial interval of 1 s followed. Each of the four experimental conditions was presented equally often (40 trials per condition). Participants performed 10 practice trials followed by an experimental block of 160 trials that were presented in a randomized order.

To quantify WM capacity we used an equation developed by Pashler [38] and modified by Cowan [9]: K = (hit rate+correct rejection rate−1)×N. This approach allows us to estimate the number of items held in memory, K, from an array size of N items, taking guessing into account. The K estimate is conceptualized as a limit in the number of discrete slots that holds a single item, which is appropriate for the change detection tasks with highly distinguishable stimuli, such as categorically different colors [39]. The K estimate has become a standard measure of change detection performance because it corrects for response bias and allows comparisons across different array sizes, conditions, and groups.

In this study, we first transformed each individual's accuracy for each array size into a K estimate. For each subject, we then calculated the mean K value across the four array sizes. The mean K values were correlated with performance in the attentional capture task (see below).

### Attentional Capture Task

#### Participants

The same PSZ participated as in the change detection task. Twenty-eight CO were matched for age (*t*_56_ = 1.38, *P* = .17), IQ (*t*_56_ = −1.33, *P* = .19), and handedness (*t*_56_ = −0.78, *P* = .44) (Table 1). PSZ were less educated than CO (*t*_56_ = −2.7, *P*<.01). 26 CO had participated in the change detection task. All subjects gave written informed consent approved by the Vanderbilt University IRB and were paid.

#### Stimuli, Task, and Procedure

Stimuli were four colored Landolt Cs (1.0°×1.0°) that appeared within placeholders (1.8°×1.8°) on a black background (Figure 1). The placeholders were present throughout the duration of each trial. The target item was a red C. The three distractors appeared in blue, magenta, or green. In each trial a search array consisting of one target and three distractors was presented for a fixed duration that was determined for each participant using a staircase procedure (see below) [15]. Participants were instructed to identify the orientation of the single target with the target color (red). Shortly following the onset of the search array, a multi-colored pattern mask was presented at each placeholder location until a response was given. Participants indicated if the gap in the target item was on the top, right, left, or bottom, by pressing one of four arrow keys. Response accuracy was emphasized. An inter-trial interval of 2 s followed. There were three types of trials, randomly intermixed. One third of the trials were flanker-present trials, i.e. at varying intervals prior to the onset of the search array a task-irrelevant colored box (flanker; 1.0°×1.0°) was presented for 50 ms at a position that flanked the position of one of the placeholders (but never the position of the target item). In two thirds of the trials, no flanker was presented. On flanker-present trials, there were four possible stimulus onset asynchronies (SOAs) between the flanker and the search array: 50 ms, 150 ms, 250 ms, and 350 ms. In half of the flanker-present trials the flanker was drawn in the target color (relevant flanker), in the other half of the flanker-present trials the flanker was green (irrelevant flanker). Participants performed 10 practice trials followed by eight experimental blocks of 120 trials, with all conditions randomly intermixed within blocks.

#### Analysis

The dependent variable was response accuracy. Because response accuracy was emphasized we did not analyze reaction time. Response accuracy was first assessed as a function of group (PSZ vs. CO) and flanker condition (no flanker, irrelevant flanker, relevant flanker) across SOAs. Because performance did not differ between PSZ and CO in the irrelevant-flanker condition (see below), the stimulus-driven effect was not further assessed.

In the relevant flanker condition when the flanker appeared in the target color, response accuracy reflects a combination of two potentially separable effects: attentional capture by the sudden onset of the flanker (stimulus-driven capture) and attentional capture by the target color (contingent capture). The contingent capture effect was isolated from the stimulus-driven effect by calculating the difference in accuracy between irrelevant-flanker trials and relevant-flanker trials ( = contingent capture cost) and compared between groups as a function of SOA

#### Staircase Procedure

Before participants performed the attentional capture task, we titrated the duration of the search array for each subject so that each subject's performance was approximately 75% correct in the no-flanker condition. In the staircase procedure, participants were initially presented with four placeholders for 500 ms. Participants were informed that a target (i.e. a red square with a gap on one side) would appear in one of the placeholders along with three distractors (i.e. differentially colored squares with a gap on one side) filling the other placeholders, and they were instructed to indicate the direction of the gap of the target with a button press. In the first trial, the target was presented for 500 ms, and thereafter, the target exposure duration was modulated from trial to trial in a following manner to obtain an individualized exposure threshold with which each individual can perform the task with approximately 75% accuracy. If a participant correctly identified the direction of the gap, the exposure duration for the next trial was shortened by 10%. On the other hand, if he/she responded incorrectly, the exposure duration for the following trial was increased by 30%. Each participant performed one practice block of 10 trials followed by four experimental blocks of 60 trials. The search-array durations for the last 20 trials in the four blocks were averaged to estimate the individual baseline search-array duration for the attentional capture task.

## Results

### Change Detection Task

The mean WM capacity averaged across the four array sizes was significantly lower for PSZ (*M* = 1.60, *SD* = 0.74, range: 0.14–3.14) than CO (*M* = 2.08, *SD* = 0.56, range: 1.26–3.4) (*t*_56_ = −2.79, *P*<.01). Lower WM capacity estimates in PSZ are consistent with past findings [1], [40]. WM capacity did not correlate with symptom ratings, chlorpromazine equivalent (CPE) dose, or duration of illness (all *P*-values >.37)

### Attentional Capture Task: Staircase Procedure

The individual baseline search-array durations were significantly longer for PSZ (*M* = 227.3, *SD* = 73.9, range: 85.3–382.3) than CO (*M* = 173.5, *SD* = 63.9, range: 67.3–316.5) (*t*_55_ = 2.91, *P*<.01). Removing one outlier from the PSZ, with an exposure time more than 3 SD higher than the group mean, did not change the result. This subject was removed from further analyses. With data collapsed across both groups (26 CO and 29 PSZ who participated in both experiments), the individual exposure time correlated negatively with the WM capacity estimate averaged across the four set sizes (*r* = −.41, *P*<.01). Trends for a similar relationship were found when the correlations were calculated separately for each group (CO, *r* = −.36, *P* = .07; PSZ, *r* = −.29, *P* = .13). The individual exposure time did not correlate with symptom ratings, CPE values, or duration of illness (all *P*-values >.72).

### Attentional Capture: Flanker Capture

**Figure 2:**
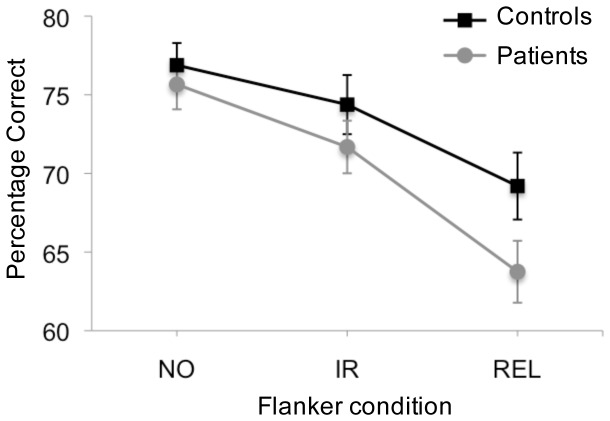
Percentage Correct as a Function of Flanker Condition and Group. (NO: no flanker, IR: irrelevant flanker, REL: relevant flanker). Chance performance is 25%. Error bars represent standard errors of the mean.

In the no-flanker condition, the mean accuracy of target identification was 76.9% for CO and 75.7% for PSZ (Figure 2). Thus, the staircase procedure was successful in equating task difficulty by individually establishing the presentation time of the search array. A repeated-measures ANOVA was conducted to examine the effects of flanker type (relevant, irrelevant, no flanker) and group (CO vs. PSZ) on accuracy. The analysis revealed a main effect of flanker type (*F*_2,110_ = 71.97, *P*<.001, *η2* = .57), with lower accuracy for irrelevant flankers than no flankers (CO, *t*_27_ = 2.78, *P*<.05; PSZ, *t*_28_ = 4.95, *p*<.001) and lower accuracy for relevant flankers than irrelevant flankers (CO, *t*_27_ = 3.64, *P*<.01; PSZ, *t*_28_ = 6.24, *P*<.001). Overall, performance did not differ between PSZ and CO (*F*_1,55_ = 1.71, *P* = .20). However, there was a significant interaction between group and flanker type (*F*_2,110_ = 3.32, *P*<.05, *η2* = .06), indicating that the additional decrease in accuracy in the relevant flanker condition was stronger in PSZ than CO. Thus, when adjusting the individual exposure time of the search array, PSZ showed a similar stimulus-driven capture effect as CO (no group difference in the irrelevant-flanker condition, *t*_55_ = −1.05, *P* = .30), whereas the contingent capture effect was slightly increased in PSZ vs. CO (trend for a significant group difference in the relevant-flanker condition, *t*_55_ = −1.85, *P* = .07).

A previous study in healthy participants demonstrated that individual differences in WM capacity were associated with the time needed to recover from attentional capture rather than the susceptibility to attentional capture [15]. To test whether PSZ and CO differed in their susceptibility to attentional capture or the time needed to recover from it, the contingent capture costs (i.e. accuracy on irrelevant-flanker trials minus accuracy on relevant-flanker trials) were compared between groups as a function of SOA. In the former case we expected PSZ to show higher capture costs than CO, irrespective of the SOA. In the latter case, capture costs should decrease faster (i.e. at earlier SOAs) in CO than PSZ. To test this hypothesis a repeated-measures ANOVA was conducted with the factors SOA [short (50-ms and 150-ms) vs. long (250-ms and 350-ms)] and group (CO vs. PSZ).

**Figure 3:**
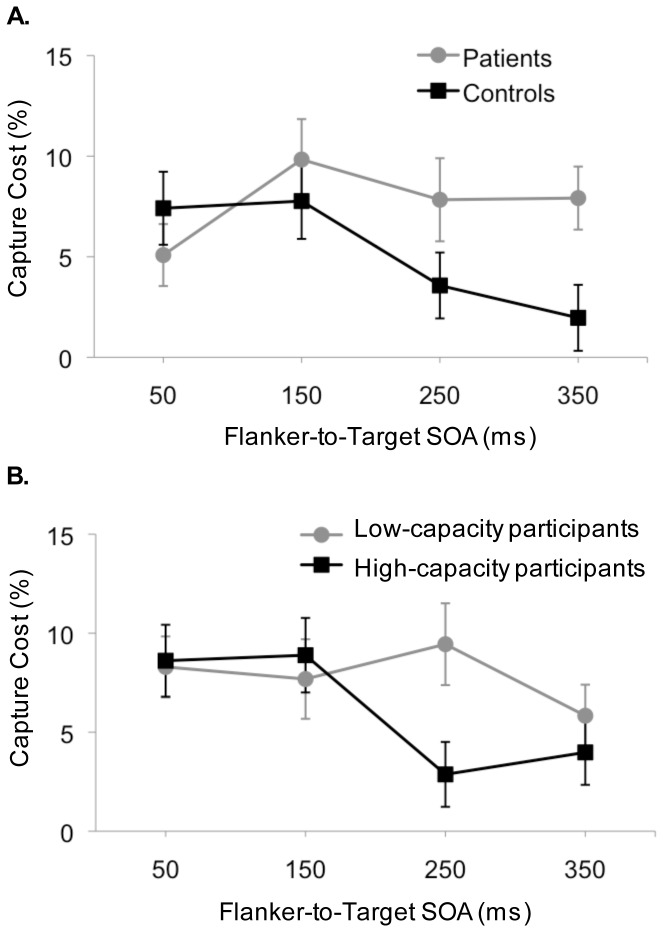
Contingent Capture Cost as a Function of Stimulus Onset Asynchrony (SOA) and Group. (A) Patients vs. Controls. (B) Low-capacity vs. High-capacity Participants. Contingent capture cost was calculated as accuracy on irrelevant-flanker trials minus accuracy on relevant-flanker trials. Error bars represent standard errors of the mean.

The analysis revealed a significant interaction effect (*F*_1,55_ = 3.99, *P* = .05, *η2* = .07) indicating equivalent capture costs in PSZ and CO for short SOAs (*t*_55_ = 0.06, *P* = .96), but a significant group difference for long SOAs (*t*_55_ = 2.27, *P*<.05). Relevant capture costs considerably decreased after the 150-ms SOA in CO, but remained high in PSZ. (see Figure 3A).

We also divided all participants into high-capacity (*M* = 2.43, *SD* = 0.39) and low-capacity groups (*M* = 1.27, *SD* = 0.45) using a median split on the WM capacity and compared contingent capture costs as a function of WM capacity (low vs. high K) and SOA (Figure 3B). This strategy followed the logic of the individual differences approach used to explore the relationship between WM and attention in healthy participants [41]. The logic is that if an individual's WM capacity can predict capture costs one can conclude that a common factor underlies both abilities. In the high-capacity group about two thirds of the participants were CO and one third of the participants were PSZ. In the low-capacity group this distribution was reversed.

Based on previous findings [15] and our findings in PSZ we predicted a faster decrease in relevant capture costs for high vs. low capacity individuals. Specifically, we expected higher capture costs in low vs. high capacity individuals for long SOAs (SOA 250-ms and 350-ms), which was tested using independent t-tests (one-tailed). The findings revealed that relevant capture costs were significantly higher in low capacity than high capacity individuals at SOA 250-ms (*t*_53_ = 2.41, *P*<.01) but did not differ between groups at SOA 350-ms (*t*_53_ = 0.81, *P* = .42).

With data collapsed across both groups, we calculated the correlation between the WM capacity estimate averaged across the four array sizes and relevant capture costs for each SOA. Although there was no relationship with WM capacity at the 50-ms and the 150-ms SOA (all *P*-values >.41), significant negative correlations emerged at SOAs 250-ms (*r* = −.33, *P*<.05) and 350-ms (*r* = −.28, *P*<.05). When calculated separately for each group, a trend for a similar relationship was found at SOA 250-ms in PSZ (*r* = −35, *P* = .06, all other *P*-values >.41) but not CO (all *P*-values >.16). Furthermore, relevant capture costs at SOA 250-ms correlated negatively with capacity estimates derived from two additional tasks, the Letter-Number-Sequencing task - reordered condition and a spatial delayed response task (DRT) with a longer encoding period [42] (see Supporting Information S1). These findings suggest that the degree to which participants, especially PSZ, are impaired in their ability to resist interference from distractors at later SOAs is related to their WM capacity reduction.

Relevant capture costs averaged across later SOAs (150–350 ms) correlated positively with SANS scores (*r* = .37, *P*<.05; *P*-values >.57 for SAPS and BPRS) but not at SOA 50-ms (*P*>.66). There was no consistent relationship with CPE values (all *P*-values >.51) and duration of illness (*r* = .41, *P*<.05 at SOA 150 ms, all other *P*-values >.46).

## Discussion

Our findings provide evidence for preserved stimulus-driven attentional capture but impaired contingent attentional capture in schizophrenia. Both groups showed equivalent capture costs when an irrelevant flanker preceded visual search, whereas capture costs were increased in PSZ compared to CO when the flanker was drawn in the target color. This new finding points to a selective impairment in the ability to resist distractor interference when top-down control [29], [30] is required to disengage from the distractor. However, when the capture is purely stimulus-driven [31], disengagement from the distractor is unimpaired in PSZ. These results are consistent with previous reports on impaired attentional selection in schizophrenia only under conditions of top-down control [19]–[21], [23], [24]. Furthermore, our results indicate that not only the process of selecting the relevant information but also the inhibition of the irrelevant information is impaired in schizophrenia.

To our knowledge only stimulus-driven attentional capture has been studied in schizophrenia so far, and the existing findings are inconclusive. Consistent, with our findings, Ducato et al. [43] did not find increased stimulus-driven attentional capture by a distractor that changed in color in PSZ. Moreover, PSZ were able to resist interference from irrelevant moving distractors to the same degree as CO when the attentional load of the central task was low [44]. However, these investigators also found increased susceptibility to attentional capture by irrelevant moving distractors in PSZ under conditions that allowed participants to control automatic capture [43]. Although these findings have been discussed in terms of domain-specific impairments in stimulus-driven attentional capture in schizophrenia, they are also consistent with the conclusion of a specific impairment in selective attention in schizophrenia when top-down processes are involved.

PSZ needed considerably more time than CO to find the target stimulus in the no-flanker condition. Thus, it was indeed crucial to adjust for differences in the processing speed when assessing attentional capture in the PSZ. When cognitive slowing was taken into account, we found no impairments in stimulus-driven capture in the PSZ. We found evidence for increased contingent capture costs in PSZ vs. CO, however these differences appeared only at later SOAs. In CO, capture costs substantially decreased at SOA 250-ms but remained consistently high in PSZ even at the latest SOA. When dividing all participants into low- and high-capacity groups we found a similar pattern of capture costs with the main difference between groups at SOA 250-ms. Because the majority of subjects in the low-capacity group were patients it is difficult to disentangle the effects of WM capacity from those related to the disease. However, Fukuda and Vogel [15] previously demonstrated in a student sample that recovery time from attentional capture was faster in individuals with high vs. low WM capacity. The faster recovery time observed in this study compared to our study, i.e. contingent capture costs substantially decreased already at SOA 150-ms in the high-capacity group, is most likely due to age differences in the study samples and/or the inclusion of patients in our high-capacity group. WM capacity decreases with age [45] and indeed, WM capacity was lower in the high-capacity group of our study than the high-capacity group in the previous study [15]. Together these findings provide evidence that recovery time rather than susceptibility to attentional capture is the critical factor that distinguishes PSZ from CO (i.e. low from high WM-capacity individuals).

The findings of this study also have important implications for an attention-based account of WM deficits in schizophrenia. We reasoned that if PSZ experienced reduced resistance to interference from distractors at an early stage of processing this would result in WM encoding problems later. To test this hypothesis, we correlated WM capacity with performance in the attentional capture task. As expected, WM capacity was markedly reduced in PSZ. The capacity estimates were lower than those reported in previous studies [22], [40], which might be due to the short stimulus presentation duration. The capacity estimates observed in the CO were also lower than the usual estimate of about 3–4 items [8], [9]. Thus, it is possible that insufficient encoding time in high WM load conditions reduced WM capacity in our participants, however it seems that this affected WM performance to a similar degree in both groups.

Consistent with an attention-based account of WM deficits in schizophrenia, we found a significant negative correlation between capacity estimates and the individual exposure time of the search array, which indicates that the degree to which participants needed more time to find the target was related to their degree of WM capacity reduction. Moreover, for relevant capture costs, there was an inverse relationship with WM capacity at later SOAs that was driven in particular by PSZ.

The observed correlations may reflect common processes other than those related to attentional selection, such as generalized cognitive impairments or reduced visual encoding. However, PSZ did not show a deficit in the irrelevant flanker condition and irrelevant capture costs did not correlate with WM capacity across all participants (all *P*-values >.26). These results do not suggest a general processing deficit but indicate a specific impairment.

Due to the short stimulus presentation time it is possible that reduced visual encoding contributed to reduced WM capacity in both groups. However, the target presentation time in the attentional capture task was individually determined. Thus, reduced encoding probably did not influence capture costs. Therefore, it seems that the common process that was impaired in both tasks and reflected in the correlation was not solely associated with visual encoding. Moreover, the group comparisons yielded a similar pattern of results as the continuous analysis showing that capture costs were considerably higher in PSZ than in CO, and higher in low- vs. high-capacity individuals at later SOAs. Finally, we demonstrated that relevant capture costs at SOA 250-ms also correlated negatively with capacity estimates derived from two additional tasks, the Letter-Number-Sequencing task and a spatial DRT that included a longer encoding period, speaking against an explanation in terms of impaired visual encoding.

In the light of previous findings that low-capacity individuals have difficulties in filtering out irrelevant information for WM encoding [11], we propose that attentional dysfunctions contribute, at least partially, to WM deficits in schizophrenia. Importantly, our findings indicate that attentional selection in the service of WM is not globally impaired in schizophrenia [22]. Rather, our data suggests that impairments in attentional selection can have detrimental effects on WM encoding specifically when top-down processes are involved. This is consistent with a previous study showing that the ability to select relevant information for WM encoding is markedly reduced in patients when the distractors have strong competitive advantage over the targets [25]. In addition to the correlational evidence derived from the present study, manipulating the demands on attentional selection during WM encoding will be necessary to further specify the attentional processes that are impaired or spared in the context of WM in schizophrenia.

Consistent with previous findings [25], our results suggest that the processes that are most vulnerable to top-down attentional dysfunctions are those required for WM encoding. However, the ability to resist interference from distractors is also crucial to keep the memory representation stable over time and to retrieve the information from WM. Thus, attentional dysfunctions are likely to affect WM at different stages of processing in schizophrenia [46].

It is important to note that recovery time from attentional capture was slowest in PSZ even though their WM capacity was higher than the estimated capacity in the low-capacity group (including PSZ and a proportion of CO) who showed no difference in capture costs compared to high capacity individuals at the latest SOA. This finding suggests dissociable effects of WM capacity and schizophrenia on contingent capture costs. It seems that the mechanisms underlying low WM capacity that might be similar in PSZ and low-capacity healthy individuals cannot fully account for the increased capture costs in the PSZ and we must consider the functional impairments that are unique to the illness.

We also found that relevant capture costs, rather than WM capacity, were associated with the severity of negative symptoms. It is unclear whether negative symptoms are more likely to be associated with reduced top-down control of attention when competition between different incoming stimuli needs to be resolved. Previous studies did not report a relationship with negative nor positive symptoms [21], [24], [25]. Attentional impairment comprises one subscale of the SANS. Although this construct is rather unspecific in the scale, its items correlate with the other negative symptom subscales [47]. Thus, it seems plausible that negative symptoms such as avolition or blunted affect lead to difficulties in the effortful allocation of attention and reflect a unique contribution of the illness to increased relevant capture costs. Given the various types of attention, this finding does not exclude the possibility that other types of attention are associated with positive symptoms.

In this study, all PSZ except for two were medicated with the majority taking second-generation antipsychotics. To assess the influence of medication, we correlated task performance with CPE. Neither WM capacity, nor the individual exposure time of the search array, or capture costs correlated with CPE. Moreover, CO with low WM capacity were medication-free and showed relevant capture costs as well. Together these findings argue against a major effect of medication on task performance.

Understanding the processes that contribute to impaired WM in schizophrenia is crucial in the search for cognitive remediation strategies. Recent studies have highlighted impairments in perceptual encoding rather than memory retention [48] and there is some evidence that theses deficits can be improved with behavioral training [49]. Our results add to these findings by suggesting that training of top-down attentional selection has the potential of enhancing WM encoding, thus pointing to different routes in the development of targeted behavioral therapies for WM deficits.

## Supporting Information

File S1 Correlations between Relevant Capture Costs and Capacity Estimates Derived from Two Additional Tasks. This file reports correlations between the relevant capture costs and capacity estimates derived from the Letter-Number-Sequencing task - reordered condition and a spatial delayed response task with a longer encoding period in patients and controls.
(DOC)
