# Supplementary material for: Impaired Contingent Attentional Capture Predicts Reduced Working Memory Capacity in Schizophrenia
Source: PLoS One. 2012 Nov 12;7(11):e48586. doi: 10.1371/journal.pone.0048586 (PMC3495971; doi:10.1371/journal.pone.0048586)
Supplement: File S1 — Correlations between Relevant Capture Costs and Capacity Estimates Derived from Two Additional Tasks. This file reports correlations between the relevant capture costs and capacity estimates derived from the Letter-Number-Sequencing task - reordered condition and a spatial delayed response task with a longer encoding period in patients and controls. (DOC) [file pone.0048586.s001.doc]

Jutta S. Mayer, Keisuke Fukuda, Edward K. Vogel, Sohee Park

**Impaired Contingent Attentional Capture Predicts Reduced Working Memory Capacity in Schizophrenia**

**Supporting Information S1.** Correlations between Relevant Capture Costs and Capacity Estimates Derived from Two Additional Tasks.

We correlated relevant capture costs with capacity estimates derived from two different tasks, the Letter-Number-Sequencing task - reordered condition [1] and a spatial delayed response task (DRT) with a longer encoding period [2].

**Letter-Number-Sequencing Task**

*Participants*

The same PSZ participated as in the change detection task and the attentional capture task. The outlier from the PSZ, with an exposure time more than 3 SD higher than the group mean, was again removed from the analysis resulting in N = 29. The same CO participated as in the attentional capture task (N = 28).

*Stimuli, Task, and Procedure*

The Letter-Number-Sequencing task – reordered condition requires the reordering of an initially unordered set of letters and numbers. The tester verbally presents increasingly longer sequences of intermixed numbers and letters (2 stimuli up to a maximum length of 7 stimuli) at a rate of 1/second. After each sequence, the participant is asked to repeat the numbers in ascending order first and then the letters in alphabetical order. Four trials at each length are presented and the test is terminated when the subject fails all four consecutive trials of the same length. Two test scores are computed; the total number of correct responses (of 24 possible) and the letter-number-span defined as the longest correctly repeated sequence.

*Results*

The mean total correct score was significantly lower for PSZ (*M* = 13.07, *SD* = 3.61, range: 2 - 20) than CO (*M* = 16.36, *SD* = 3.18, range: 11 - 23) (*t*_55_ = -3.64, *P* < .01). Similarly, the letter-number-span was lower for PSZ (*M* = 5.21, *SD* = 1.01, range: 2 - 7) than CO (*M* = 5.89, *SD* = 0.99, range: 4 - 7) (*t*_55_ = -2.58, *P* < .05).

Across both groups, the letter-number-span and the total correct score correlated negatively with relevant capture costs at SOA 250-ms (*r* = .-33, *P* < .05, *r* = .-34, *P* < .05, respectively), but not at any other SOA (all *P*-values *>* .17). When calculated separately for each group, trends for a similar relationship were found at the SOA 250-ms in PSZ (letter-number-span, *r* = .-32, *P* = .09, total score, *r* = .-35, *P* = .06) but not CO (all *P*-values > .30).

**Spatial Delayed Response Task (DRT)**

*Participants*

25 PSZ and 22 CO who participated in the attentional capture task also performed the spatial DRT.

*Stimuli, Task, and Procedure*

The stimuli, task, and procedure are described in detail in Mayer et al. [2]. Participants were presented with either one target (the letter A, presented either upright or upside-down) or a sequence of three targets. The targets appeared at different positions on the screen and participants were asked to encode their positions into WM. Each target stimulus was presented for a duration of 750ms and separated by an inter-stimulus interval of 250 ms. After an 8s delay interval, a question mark was presented as a probe until a response was given. Participants indicated whether the position of the question mark matched one of the target positions by a left or right key press for match and non-match, respectively. Half of the trials were matches. See Figure 1 in Mayer et al. [2] for an illustration of the sequence of events in each trial.

For the upright condition, each individual’s response accuracy was transformed into a K estimate using the formula proposed by Cowan [3]. For each subject, the values for WM load 1 and 3 were averaged into a single WM capacity estimate.

*Results*

The mean WM capacity estimate was significantly lower for PSZ (*M* = 0.51, *SD* = 0.46, range: -0.19 – 1.32) than CO (*M* = 1.0, *SD* = 0.55, range: -0.12 – 2.0) (*t*_45_ = -3.33, *P* < .001). Across both groups, WM capacity estimates correlated negatively with relevant capture costs only at SOA 250–ms (*r* = .-40, *P* < .01, all other *P*-values > .06). A similar relationship was found for CO (SOA 250-ms, *r* = .-53, *P* < .05, all other *P*-values > .12) but not PSZ (all *P*-values > .09) when calculated separately for each group.

**References**

1. Gold JM, Carpenter C, Randolph C, Goldberg TE, Weinberger DR (1997) Auditory working memory and Wisconsin Card Sorting Test performance in schizophrenia. Arch Gen Psychiatry 54: 159-165.
2. Mayer JS, Kim J, Park S (2011) Enhancing visual working memory encoding:

The role of target novelty. Vis Cogn 19: 863-885.

1. [Cowan N](http://www.ncbi.nlm.nih.gov/pubmed?term="Cowan N"%5BAuthor%5D) (2001) The magical number 4 in short-term memory: a reconsideration

of mental storage capacity. [Behav Brain Sci](javascript:AL_get(this, 'jour', 'Behav Brain Sci.');) 24: 87-114.
